# Validation of a Biofeedback System for Wheelchair Propulsion Training

**DOI:** 10.1155/2011/590780

**Published:** 2011-10-05

**Authors:** Liyun Guo, Andrew M. Kwarciak, Russell Rodriguez, Nilanjan Sarkar, W. Mark Richter

**Affiliations:** ^1^Biomechanics Laboratory, MAX Mobility, LLC, 5425 Mount View Parkway, Antioch, TN 37013, USA; ^2^Department of Mechanical Engineering, Vanderbilt University, Nashville, TN 37235, USA

## Abstract

This paper describes the design and validation of the OptiPush Biofeedback System, a commercially available, instrumented wheel system that records handrim biomechanics and provides stroke-by-stroke biofeedback and targeting for 11 propulsion variables. Testing of the system revealed accurate measurement of wheel angle (0.02% error), wheel speed (0.06% error), and handrim loads. The maximum errors in static force and torque measurements were 3.80% and 2.05%, respectively. Measured forces were also found to be highly linear (0.985 < slope < 1.011) and highly correlated to the reference forces (*r* ^2^ > .998). Dynamic measurements of planar forces (*F*_*x*_ and *F*_*y*_) and axle torque also had low error (−0.96 N to 0.83 N for force and 0.10 Nm to 0.14 Nm for torque) and were highly correlated (*r* > .986) with expected force and torque values. Overall, the OptiPush Biofeedback System provides accurate measurement of wheel dynamics and handrim biomechanics and may be a useful tool for improving manual wheelchair propulsion.

## 1. Introduction

As the study of manual wheelchair propulsion has progressed over the past few decades, so have the tools used to assess and improve propulsion. Early investigations of wheelchair propulsion focused on physiologic energy costs [1–3] using common medical equipment such as spirometers. In 1976, Brubaker and Ross [4] performed one of the first investigations of wheelchair handrim loading using a custom-made test stand. The stand included an instrumented beam that could measure tangential force applied to the handrim. Other early measurements of handrim kinetics involved similar, instrumented fixtures [5, 6]. The continued advancement of wheelchair propulsion research, which included the incorporation of motion capture technology [7] and inertial roller systems [8, 9], led to the development of the self-contained, instrumented wheel [10–13]. 

Several research groups have developed instrumented wheel systems that can measure three-dimensional handrim forces and torques [11–13]. Two systems [12, 13] were developed around a commercial, 6 degree-of-freedom load cell. In both designs, the load cell is mounted to the center of the wheel, and an interface plate attaches the handrim to the load cell. The third system, the SmartWheel, uses an array of strain gauges, bonded to three beams connecting the handrim to the wheel, and an optical encoder to measure handrim kinetics as well as wheel angle and speed [10, 11]. Developed by Dr. Rory Cooper and his colleagues at the University of Pittsburgh, the SmartWheel is commercially available through Three Rivers Holdings, LLC (Mesa, AZ, USA) and has been used by a number of researchers and clinicians to study manual wheelchair propulsion and use [14–17]. 

While each instrumented wheel system has demonstrated good accuracy and linearity, as well as the ability to measure typical propulsion forces and torques, they are limited in their ability to improve propulsion technique. None of the systems provide real-time feedback on selectable variables in an easily interpretable format. The SmartWheel software can display real-time plots of tangential force and speed along with a set of calculated variables including peak force, push length, and cadence; however, the display cannot isolate variables or show performance targets, making it difficult for users to understand what and how much they should improve. The limitations of instrumented wheels are also attributed to their fixed wheel diameters. The SmartWheel is available in multiple sizes, ranging from 22′′ to 26′′, but each size requires the purchase of an additional wheel. Researchers, clinicians, and users can benefit from a more versatile tool that promotes improvements in wheelchair propulsion biomechanics.

Expanding upon previous systems, we developed a new instrumented wheel system, named the OptiPush Biofeedback System, to measure handrim biomechanics and provide real-time biofeedback for a wide variety of wheelchair users. The system was designed to study propulsion biomechanics and help train users to improve their propulsion technique. As the OptiPush has gained popularity with clinicians and researchers, it is important to detail its design and measurement accuracy. The purpose of this study was to (1) describe the design of the OptiPush system and (2) validate OptiPush measurements of wheel angle, speed, and three-dimensional handrim forces and torques.

## 2. Materials and Methods

### 2.1. Mechanical Design

**Figure 1:**
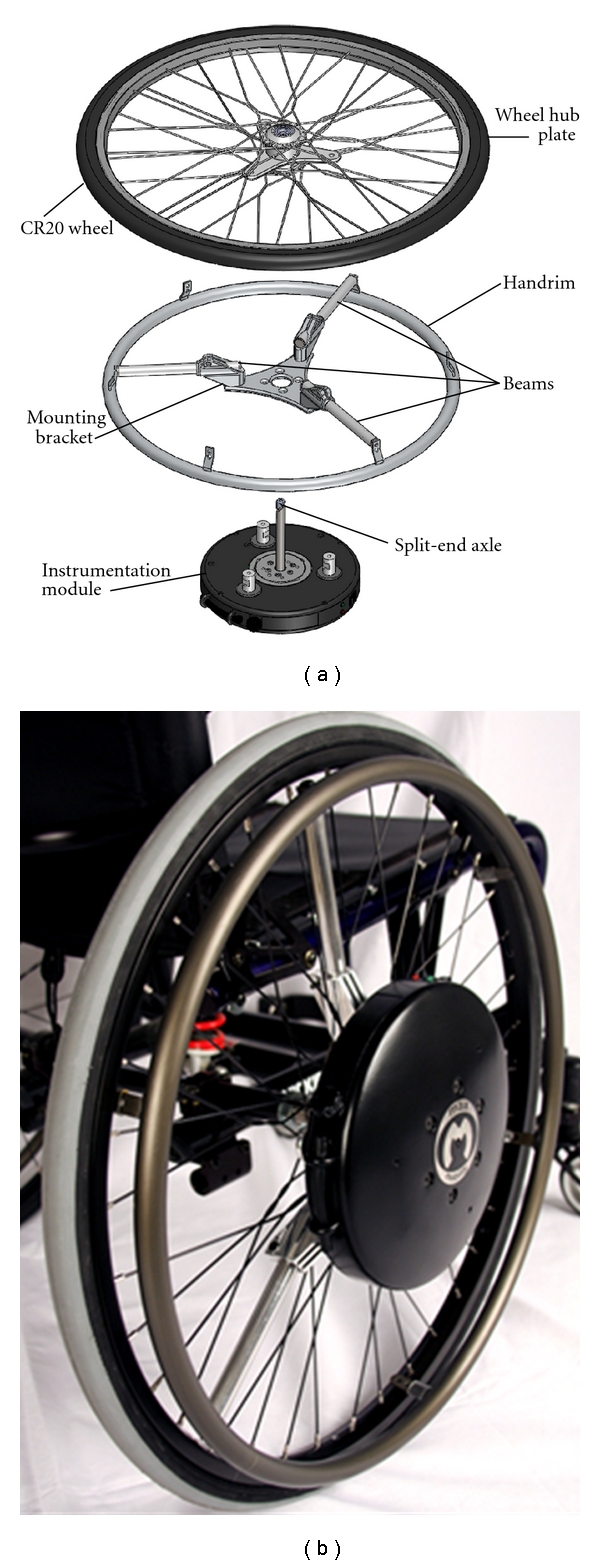
The components (a) and assembly (b) of the OptiPush wheel.

The OptiPush wheel (Figure 1) is composed of a Sun CR20 wheel (Sun Components, Milwaukee, WI, USA), with a modified hub, a handrim, a mounting bracket, three aluminum beams, and an instrumentation module (IM). The IM houses the sensors and electrical components of the device (described in the following section). To measure the loads applied to the handrim, the IM is connected directly to the handrim through the mounting bracket and beams. The length of beam protruding from the bracket can be adjusted to accommodate different handrim sizes. The outer end of each beam is screwed to one of three modified handrim tabs. The IM-handrim assembly is mounted to the wheel by screwing the IM directly to wheel hub plate. This modular instrumentation design allows the system to incorporate different sized wheels (20′′, 22′′, 24′′, 25′′, and 26′′ diameter). For a 25′′ (559 mm) wheel, the total mass of the OptiPush wheel is 6.0 kg. Once assembled, the wheel is mounted to the wheelchair by tightening the split-end axle in the axle receiver.

### 2.2. Electrical Design

The OptiPush wheel measures handrim loads using a commercially available 6 degree-of-freedom load cell (Delta; ATI Industrial Automation, Apex, NC, USA). The load cell has full-range mechanical load limit of 770 Newtons (N) for forces in the plane of the wheel (*F*_*x*_ and *F*_*y*_), 2310 N for forces perpendicular to the plane of the wheel (*F*_*z*_), and 70 Newton-meters (Nm) for torques about all three axes. An absolute rotary encoder (MA3; US Digital, Vancouver, WA, USA) is used to measure wheel angle. The encoder reports shaft position continuously over 360° without gaps. A Bluetooth module (BlueSentry RN-800S; Roving Networks, Inc., Los Gatos, CA, USA), with an 8 channel, 16 bit analog-to-digital converter samples load cell and encoder signals and converts them to a Bluetooth-enabled digital data stream. The data stream is received (using a Bluetooth dongle) and recorded by a designated computer running the OptiPush software. All components within the OptiPush wheel are powered by a 7.4 V 2600 mAh Li-ion rechargeable battery, which can provide power for more than three hours before recharging. Two voltage regulators create the required voltage for each component.

### 2.3. OptiPush Software

**Figure 2:**
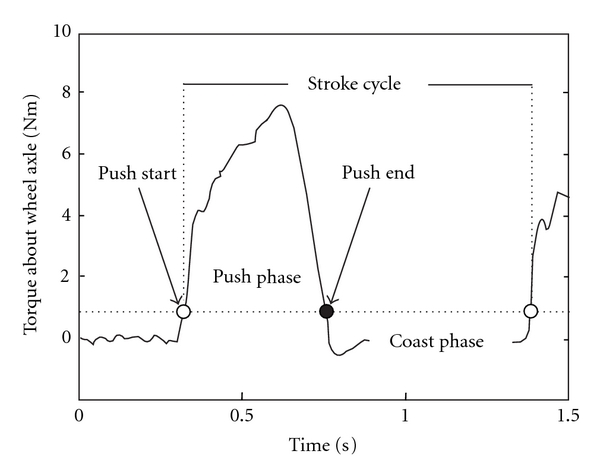
Definition of the stroke cycle, push phase, and coast phase.

The OptiPush Software records, saves, and displays data from the OptiPush wheel. Data are sampled at 200 Hz and filtered with a fourth-order Butterworth digital low-pass filter with a 20 Hz cutoff frequency [18]. Measurements from a setup trial are used to determine the dynamic offset of each load cell channel. As data are sampled, the dynamic offsets are removed and the load cell calibration matrix is applied to the raw voltages, resulting in conditioned force and torque outputs [19]. Data are segmented into stroke cycles based on absolute torque about the wheel axle (*T*_*z*_). A stroke cycle begins with the push phase, the period when *T*_*z*_ exceeds 1 Nm, and ends with the recovery (or coast) phase, the period when *T*_*z*_ is below 1 Nm (Figure 2). 

**Figure 3:**
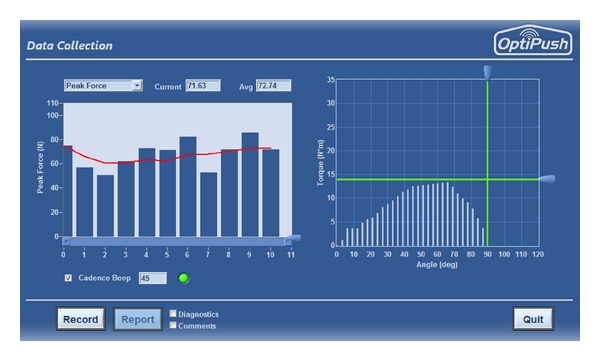
Example of the push-by-push biofeedback display.

The OptiPush Software includes multivariable biofeedback, a novel addition to instrumented wheelchair wheel technology. Using force, torque, and wheel angle data, the software calculates eleven biofeedback variables: braking torque, cadence, coast time, contact angle, impact, peak force, peak torque, power output, push distance, smoothness, and speed (Table 1). Each variable can be displayed in a bar graph format (Figure 3) with a running average of the last 5 strokes. A target value can be set to help wheelchair users reach or maintain a desired value. For cadence, an auditory beep is also available. The efficacy of this biofeedback has been proven in a separate study, in which subjects were able to use the biofeedback to make significant and targeted improvements to several propulsion metrics [20].

### 2.4. System Validation

Static and dynamic tests were conducted to validate the ability of the OptiPush Biofeedback System to accurately measure wheel angle, speed, and handrim forces and torques. For all dynamic tests, the OptiPush wheel was attached to the right side of a Quickie XTR wheelchair (Sunrise Medical, Longmont, CO), and the wheelchair was attached to a motor-driven, wheelchair accessible treadmill [21]. The selection of the right side was arbitrary. The validity of the system is assumed to be independent of mounting side. Two safety straps were attached to the front frame of a wheelchair to prevent it from veering off the belt or tipping over backwards. 

### 2.5. Wheel Angle Measurement

The treadmill was set to run at a constant speed of about 0.7 m/s. The revolutions of the wheel (effective diameter: 635 mm) were counted manually while the OptiPush Software recorded wheel angle. The treadmill was stopped after 100 revolutions had been counted. Wheel orientations at both the start and stop positions were measured. The wheel angle measured by the OptiPush Software was compared with wheel angle calculated from the wheel revolutions.

### 2.6. Speed Calculation

OptiPush wheel speed is calculated by multiplying angular speed by wheel diameter; therefore, validation required determining the accuracy of both angular speed (the rate of change of wheel angle) and wheel diameter. Given the previous validation of wheel angle, the accuracy of the OptiPush speed calculations were based on the variability in experimental calculations of wheel diameter for each of the five different OptiPush wheel sizes (508 mm, 559 mm, 610 mm, 635 mm, and 660 mm effective diameter). All tires were inflated to their recommended tire pressure of 758 kilopascals (110 psi). To simulate typical conditions, an 85 kg adult male sat in the wheelchair, which was secured to the treadmill. The treadmill speed was set to approximately 1 m/s and run for 30 seconds. During the trial, the revolutions of the treadmill belt and OptiPush wheel were counted. Two trials were conducted for each wheel size. Using both sets of revolutions and the treadmill belt length, wheel diameter (*D*) was calculated as

(1) $$D=\frac{\text{Revolutions}\;\text{of}\;\text{belt}\;\times \;\text{belt}\;\text{length}}{\text{Revolutions}\;\text{of}\;\text{wheel}},$$

where belt length was 5.69 m. The error in wheel diameter was then determined as the percent difference between the two calculations.

### 2.7. Force and Torque Measurement

The forces along the fore-aft (*F*_*x*_) and superior-inferior (*F*_*y*_) axes and the torque about the medial-lateral (*T*_*z*_) axis were validated with both static and dynamic tests, while the force along the medial-lateral (*F*_*z*_) axis and the torques about the fore-aft (*T*_*x*_) and superior-inferior (*T*_*y*_) axes were validated with just static tests. For each test, the forces and torques measured by the OptiPush were compared against the weight, position, and movement (for dynamic tests) of the attached load. 

#### 2.7.1. Static Tests

To test *F*_*x*_, *F*_*y*_, and *T*_*z*_, the OptiPush wheel was positioned vertically, in the standard wheelchair position. Three reference loads (23.28 N, 68.04 N, and 109.99 N), similar to those used previously [13], were hung from the bottom of the handrim at eight different wheel angles (0°–315° in 45° increments) such that the resultant force in the plane of the wheel (√[*F*_*x*_^2^ + *F*_*y*_^2^]) should equal the weight of each load. The two smaller reference loads (23.28 N and 68.04 N) were also hung on each of the three beams at the point of attachment to the handrim (such that the load radius equaled the handrim radius). Before the load was applied, the beam was horizontally leveled so *T*_*z*_ could be calculated as the weight of load multiplied by radius of handrim. To test *F*_*z*_, *T*_*x*_, and *T*_*y*_, the OptiPush wheel was positioned horizontally with the handrim facing upwards. The 23.28 N, 68.04 N, and 109.99 N loads were hung from each of the three beams to generate values of *F*_*z*_. The 23.28 N and 68.04 N loads were also hung from the handrim such that the combined torque (√[*T*_*x*_^2^ + *T*_*y*_^2^]) should equal the weight of each load multiplied by the radius of the handrim. Each static test lasted about 10 seconds.

#### 2.7.2. Dynamic Tests

**Figure 4:**
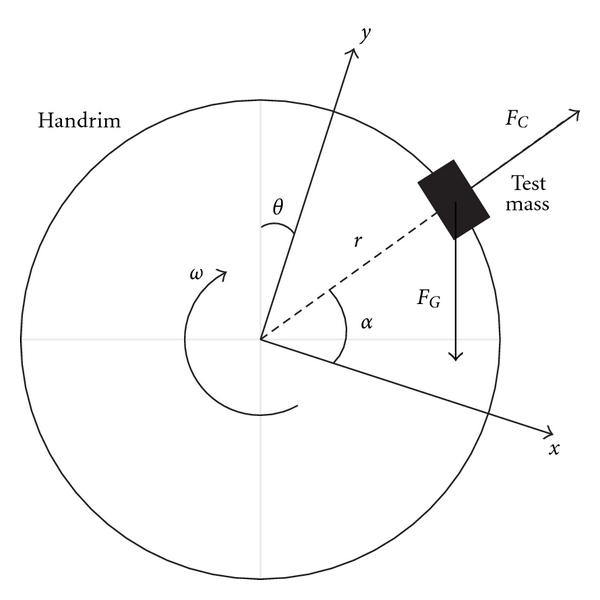
Centrifugal force (*F*_*C*_) and gravitation (*F*_*G*_) force of the test mass applied to the handrim during dynamic testing, where *θ* is the wheel angle, *ω* is the angular velocity of the wheel, *r* is the radius of the handrim, and *α* is the angle of the mass with respect to the load cell coordinate system.

Dynamic tests were done to further validate *F*_*x*_, *F*_*y*_, and *T*_*z*_ under more realistic testing conditions. One at a time, two masses (1.17 kg and 2.30 kg) were secured to the handrim. For each mass, the treadmill was run at three different speeds (0.5 m/s, 1.0 m/s, and 1.5 m/s) for at least 10 wheel revolutions. As the wheel rotated on the treadmill belt, the attached mass applied a downward (gravitational) and outward (centrifugal) force to the handrim (Figure 4). The centrifugal forces in the plane of the wheel (*F*_*x*_ and *F*_*y*_) and torque about the wheel axle (*T*_*z*_) were calculated with the following equations:

(2) $$\begin{array}{c} F_{x}=F_{G}\cdot \sin\;\left(\theta \right)+m\cdot \omega^{2}\cdot r\cdot \cos\;\left(\alpha \right),\\ F_{y}=-F_{G}\cdot \cos\;\left(\theta \right)+m\cdot \omega^{2}\cdot r\cdot \sin\;\left(\alpha \right),\\ T_{z}=F_{G}\cdot \cos\;\left(\alpha -\theta \right)\cdot r, \end{array}$$

where *θ* is the wheel angle; *ω* is the angular velocity of the wheel; *r* is the radius of the handrim; *m* is the added mass, and *α* is the angle of the mass. Since the values of *F*_*x*_ and *F*_*y*_ could be zero during testing, the errors were presented as the differences between the measured and calculated values. Pearson product-moment correlations were performed to assess the relationship between the values of *F*_*x*_, *F*_*y*_, and *T*_*z*_.

## 3. Results

### 3.1. Wheel Angle and Speed Validation

Treadmill testing revealed good wheel angle and wheel speed accuracy. The OptiPush was able to measure wheel angle to within 0.02% of the total angle. For the diameter test, the absolute error in wheel diameter was no greater than 0.04%, resulting in a maximum wheel speed error of 0.06%.

### 3.2. Force and Torque Validation

Tables 2, 3, and 4 show the measured force for each static test and their percent difference (error) from the actual values. Overall, the maximum absolute error in force was 3.8%, and the maximum absolute error in torque was 2.04%. Measurements of force in all three directions were highly linear (0.985 < slope < 1.011) and highly correlated to the actual force values (*r* ^2^ > 0.998).  Table 5 shows the results of the dynamic force and torque tests. The measured values of *F*_*x*_, *F*_*y*_, and *T*_*z*_ were highly correlated with the actual forces (*r* > 0.986; *P* < 0.05). The mean errors in force ranged from −0.96 N to 0.83 N (maximum standard deviation of 1.55 N), and the mean errors in torque ranged from 0.10 Nm to 0.14 Nm (maximum standard deviation of 0.35 Nm).

## 4. Discussion

In a series of static and dynamic tests, the OptiPush provided accurate measurements of wheel angle, speed, and the forces and torques applied to the handrim. The errors in wheel angle and wheel speed were both less than 0.1%. These values are well within the expected variability in contact angle and speed measured within and across push strokes [15]. While the accuracy of both wheel angle and speed are dependent on the accuracy of the rotary encoder, it is important to experimentally confirm the final computations. The only other instrumented wheel to include a rotary encoder is the SmartWheel, for which wheel angle and speed measurement accuracies are unavailable.

For force and torque measurements, the maximum errors were 3.8% and 2.04%, respectively. These errors are within the factory-determined error of the load cell (1.5% of the full-scale load limit of each axis) and comparable to those of other instrumented wheels [11, 13]. The fact that force error was larger than torque error was expected, as the full-range load limits for force measurement (770 N for *F*_*x*_ and *F*_*y*_; 2310 N for *F*_*z*_) are larger than the load limit for torque measurement (70 Nm). The linearity was also high and consistent with previous systems [11–13]. In dynamic tests, the measurements of *F*_*x*_, *F*_*y*_, and *T*_*z*_ were all highly correlated with the calculated values. The errors in each measure were small and may be attributed to treadmill vibrations and/or inaccuracies in calculating the effect of the reference masses. Due to difficulties in measuring the weight center of the mass attached to the handrim, there may have been errors in the relative angles of mass. While these errors could have affected the accuracy in calculating the reference force and torque values, they were not large enough to warrant further investigation.

## 5. Conclusions

The modular design of the OptiPush wheel and the accuracy with which it can measure wheel angle, speed, and handrim forces and torques make the OptiPush Biofeedback System an effective tool for assessing handrim biomechanics. Future investigations will test the effects of multivariable biofeedback and develop training protocols to improve propulsion biomechanics.

## Figures and Tables

**Table 1:** Description of biofeedback variables.

Variable	Description (Units)
Braking torque	Maximum negative (braking) torque about the axle within the stroke (Nm)
Cadence	Push frequency (pushes/min)
Coast time	Time between handrim release and subsequent handrim contact (s)
Contact angle	Angle through which wheel rotates when hand is contact with handrim (degrees)
Impact	Maximum rate of force applied to the handrim (N/s)
Peak force	Maximum total force during the stroke, where total force = √(*F*_*x*_^2^ + *F*_*y*_^2^ + *F*_*z*_^2^) (N)
Peak torque	Maximum torque about the axle during the stroke (Nm)
Power output	Power generated by applying a torque about the axle for a given contact angle (W)
Push distance	Distance travelled during the stroke (m)
Smoothness	Mean force divided by peak force, unit-less
Speed	Mean speed during the stroke (m/s)

N: Newton; m: meter; W: Watts.

**Table 2:** Validation of *F*_*xy*_ measurements.

Wheel angle (degrees)	Actual force = 23.28 N		Actual force = 68.04 N		Actual force = 109.99 N	
	Measured force (N)	Error (%)	Measured force (N)	Error (%)	Measured force (N)	Error (%)
0	23.11 ± 1.59	−0.74	66.55 ± 1.65	−2.19	108.68 ± 1.47	−1.19
45	23.75 ± 1.66	2.02	67.05 ± 1.74	−1.45	109.17 ± 1.47	−0.75
90	22.94 ± 1.21	−1.48	66.26 ± 1.53	−2.62	108.25 ± 1.35	−1.59
135	24.04 ± 1.63	3.24	67.39 ± 1.35	−0.96	109.45 ± 1.44	−0.49
180	23.85 ± 1.47	2.43	67.08 ± 1.50	−1.42	109.23 ± 1.70	−0.69
225	23.38 ± 1.68	0.41	66.65 ± 1.75	−2.05	108.92 ± 1.59	−0.97
270	22.75 ± 1.34	−2.28	65.84 ± 1.42	−3.23	107.78 ± 1.41	−2.01
315	22.40 ± 1.89	−3.80	65.51 ± 1.64	−3.72	107.43 ± 1.57	−2.33

Measured values are mean ± 1 standard deviation.

**Table 3:** Validation of *F*_*z*_ measurements.

Actual force (N)	Measured force (N)	Error (%)
23.28	23.31 ± 3.20	0.10
68.04	66.49 ± 3.74	−2.28
109.99	112.66 ± 3.16	2.42

Measured values are mean ± 1 standard deviation.

**Table 4:** Validation of torque measurements.

Variable	Actual torque (Nm)	Measured torque (Nm)	Error (%)
√(*T*_*x*_^2^ + *T*_*y*_^2^)	6.03	5.99 ± 0.09	−0.56
	17.61	17.45 ± 0.10	−0.89
*T*_*Z*_	6.03	6.08 ± 0.07	0.87
	17.61	17.97 ± 0.07	2.04

Measured values are mean ± 1 standard deviation.

**Table 5:** Results of dynamic testing.

		Test Mass = 1.17 kg		Test Mass = 2.30 kg	
Variable	Speed (m/s)	Correlation coefficient	Measured value-actual value	Correlation coefficient	Measured value-actual value
*F*_*x*_ (N)	0.5	0.989	−0.04 ± 1.20	0.998	0.11 ± 1.31
	1.0	0.994	0.31 ± 0.93	0.998	0.83 ± 1.22
	1.5	0.994	0.42 ± 0.92	0.997	0.69 ± 1.36
*F*_*y*_ (N)	0.5	0.988	−0.20 ± 1.32	0.997	−0.28 ± 1.25
	1.0	0.993	−0.60 ± 1.03	0.998	−0.96 ± 1.03
	1.5	0.993	−0.81 ± 1.16	0.995	−0.60 ± 1.55
*T*_*z*_ (Nm)	0.5	0.999	0.13 ± 0.08	0.999	0.14 ± 0.11
	1.0	0.997	0.13 ± 0.17	0.998	0.14 ± 0.26
	1.5	0.986	0.11 ± 0.35	0.998	0.10 ± 0.25

Differences in the values are mean ± 1 standard deviation.
